# Accuracy of Mixed Reality Combined With Surgical Navigation Assisted Oral and Maxillofacial Tumor Resection

**DOI:** 10.3389/fonc.2021.715484

**Published:** 2022-01-14

**Authors:** Zu-Nan Tang, Lei-Hao Hu, Hui Yuh Soh, Yao Yu, Wen-Bo Zhang, Xin Peng

**Affiliations:** ^1^ Department of Oral and Maxillofacial Surgery, Peking University School and Hospital of Stomatology & National Center of Stomatology & National Clinical Research Center for Oral Diseases & National Engineering Research Center of Oral Biomaterials and Digital Medical Devices, Beijing, China; ^2^ Department of Oral and Maxillofacial Surgery, Faculty of Dentistry, Universiti Kebangsaan Malaysia, Kuala Lumpur, Malaysia

**Keywords:** virtual surgical plan, mixed reality, surgical navigation technique, oral and maxillofacial tumor, jaw surgery

## Abstract

**Objective:**

To evaluate the feasibility and accuracy of mixed reality combined with surgical navigation in oral and maxillofacial tumor surgery.

**Methods:**

Retrospective analysis of data of seven patients with oral and maxillofacial tumors who underwent surgery between January 2019 and January 2021 using a combination of mixed reality and surgical navigation. Virtual surgical planning and navigation plan were based on preoperative CT datasets. Through IGT-Link port, mixed reality workstation was synchronized with surgical navigation, and surgical planning data were transferred to the mixed reality workstation. Osteotomy lines were marked with the aid of both surgical navigation and mixed reality images visualized through HoloLens. Frozen section examination was used to ensure negative surgical margins. Postoperative CT datasets were obtained 1 week after the surgery, and chromatographic analysis of virtual osteotomies and actual osteotomies was carried out. Patients received standard oncological postoperative follow-up.

**Results:**

Of the seven patients, four had maxillary tumors and three had mandibular tumors. There were total of 13 osteotomy planes. Mean deviation between the planned osteotomy plane and the actual osteotomy plane was 1.68 ± 0.92 mm; the maximum deviation was 3.46 mm. Chromatographic analysis showed error of ≤3 mm for 80.16% of the points. Mean deviations of maxillary and mandibular osteotomy lines were approximate (1.60 ± 0.93 mm vs. 1.86 ± 0.93 mm). While five patients had benign tumors, two had malignant tumors. Mean deviations of osteotomy lines was comparable between patients with benign and malignant tumors (1.48 ± 0.74 mm vs. 2.18 ± 0.77 mm). Intraoperative frozen pathology confirmed negative resection margins in all cases. No tumor recurrence or complications occurred during mean follow-up of 15.7 months (range, 6-26 months).

**Conclusion:**

The combination of mixed reality technology and surgical navigation appears to be feasible, safe, and effective for tumor resection in the oral and maxillofacial region.

## Introduction

The oral and maxillofacial region is anatomically complex, housing many vital vessels and major nerves. Because tumors in this region are often deep seated, resection surgery can be challenging. Thorough understanding of the tumor site and margins, individualized surgical planning, and accurate implementation of the surgery are of paramount importance (1). Recent advances in computer-assisted surgery (CAS), particularly in three-dimensional (3D) reconstruction, virtual surgical planning, and surgical navigation, have greatly improved the safety and accuracy of surgery in the maxillofacial region (2, 3). Surgical navigation offers real-time visual feedback and greatly facilitates implementation of the virtual surgical plan, but it does have limitations. Pietruski et al. (4) noted that constant gazing at the monitor screen adversely affects the surgeon’s hand–eye coordination and, thereby, surgical efficiency and accuracy. Moreover, despite continuing improvements in surgical navigation (5), the 3D image display remains two-dimensional, and accurate projection of the images to the surgical field still depends on the surgeon’s experience and imagination (6).

Mixed reality is an emerging holographic technology that combines the advantages of virtual reality and augmented reality. Image processing and mathematical computation is used to generate and project a real-time 3D hologram with which the user can interact. Mixed reality technology has been applied in the fields of hepatobiliary surgery and neurosurgery, but its application in oral and maxillofacial surgery remains limited.

In the maxillofacial region, satisfactory functional and aesthetic reconstruction is as important as accurate and safe tumor ablation. With mixed reality technology it is possible to project a 3D hologram on to the surgical field, but accurate registration is difficult. With surgical navigation, however, it is possible to achieve satisfactory registration accuracy. We hypothesized that the combination of mixed reality and surgical navigation could be used for safe and accurate resection of tumors located in the oral and maxillofacial region, and have applied the technique at our hospital. The aim of this retrospective study was to describe the technique and evaluate its feasibility and accuracy in oral and maxillofacial tumor surgery.

## Materials and Methods

### Patients

A total of seven patients diagnosed with oral and maxillofacial tumors in the Department of Oral and Maxillofacial Surgery, Peking University School of Stomatology between January 2019 and January 2021 were included in this study. All seven patients 1) had diagnosis confirmed by preoperative incisional biopsy; 2) had tumor involving both hard and soft tissues of the oral and maxillofacial region and required maxillectomy or mandibulectomy; 3) had no absolute contraindication for surgery; and 4) received treatment planning and tumor resection with the combination of mixed reality and surgical navigation.

This study was approved by the Institutional Review Board of Peking University School of Stomatology (approval number: PKUSSIRB-202054028). Written informed consent was obtained from all patients preoperatively.

### Multimodal Image Fusion

**Figure 1:**
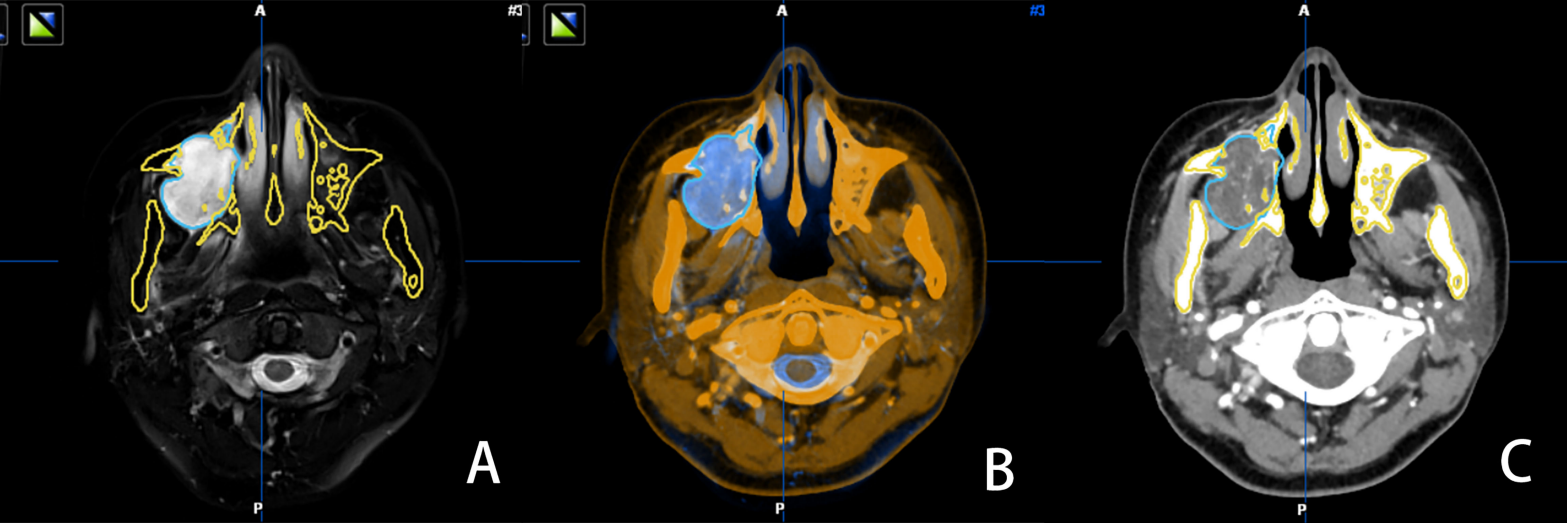
MRI image, with tumor boundary depicted in blue **(A)** the software automatically recognized the bone tissue structure of the CT and MRI images in the same slice and fused them **(B)** the tumor boundary is marked in blue in the CT image **(C)**.

All patients were subjected to standard preoperative head and neck computed tomography (CT) scan (field of view, 20 cm; pitch, 1.0; slice thickness, 1.25 mm; 140-160 mA, pixel density, 512 × 512); and magnetic resonance imaging (MRI; T2-weighted sequence, 1.5T (1T = 800 kA/m); slice thickness, 2 mm; pixel density, 512 × 512). Patients were required to maintain full intercuspal position during imaging. Digital Imaging and Communication in Medicine (DICOM) data of CT and MRI were uploaded to iPlan CMF 3.0 software (BrainLAB, Feldkirchen, Germany). Image fusion was performed using automatic fusion technique, with the tumor set as the region of interest (ROI) in both datasets. After accurate alignment of CT and MRI images at each slice, tumor mapping was performed on the MRI dataset (**Figure 1A**). The iPlan CMF 3.0 software enabled automatic registration of the two datasets, with bony structures as the references (**Figure 1B**). Finally, the mapped tumor margins on MRI were projected on to the CT datasets (**Figure 1C**). Using the multimodal image fusion technique, tumor margins were mapped and the surgical margins were planned virtually to ensure safe surgical margins during the actual surgery.

### Virtual Surgery Design

**Figure 2:**
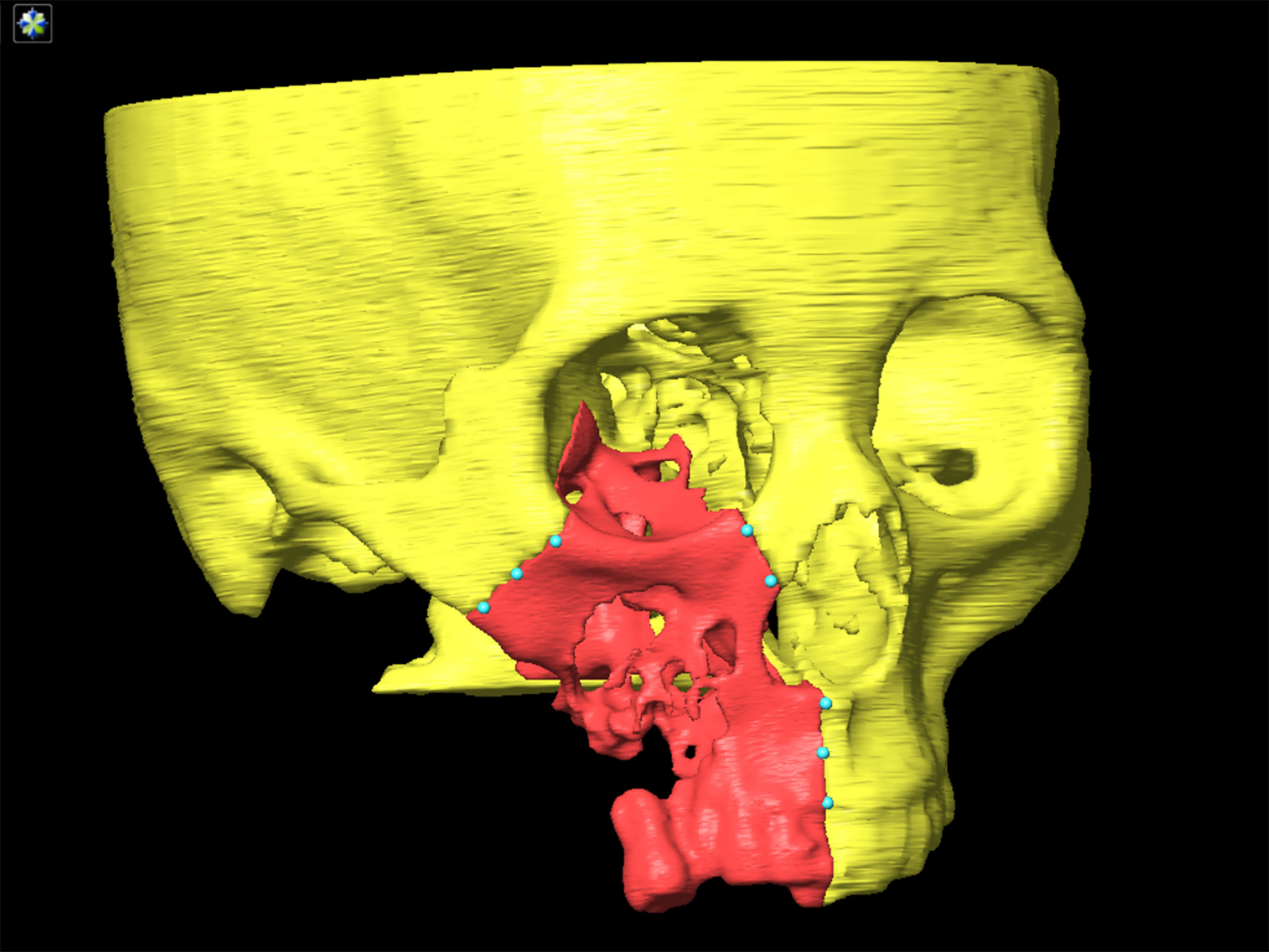
More than two points are marked on the designed osteotomy line.

Following image fusion, the datasets were imported into a virtual surgical planning software (ProPlan CMF 3.0; Materialise, Belgium) for planning of osteotomy planes. The preoperative plan was designed under the cooperation of a well-experienced oral and maxillofacial surgeon and an experienced biomedical engineer. For each osteotomy plane, two reference points were marked manually (**Figure 2**). The virtual surgical plan was exported in STL format into the navigation workstation (VectorVision, Brainlab, Germany) for intraoperative navigation.

### Mixed Reality and Navigation Connection Registration

Under general anesthesia, a 1-cm scalp incision was made. Then, following fixation of dynamic reference frame, laser surface scanning was used for skin surface registration. Synchronization of mixed reality and surgical navigation was completed using the IP address of surgical navigation *via* IGT-link port connection with both workstations. Open IGT-link is a network protocol that allows network communication among medical devices and supports image data transfers, based on the agreement by National Alliance for Medical Image Computing (NA-MIC). The devices (including tablet and camera) were connected to the mixed reality workstation (Visual3d, China) through the local network portal. Image data from the surgical navigation workstation was cached in the mixed reality workstation prior to its projection to the head-mounted mixed reality device, HoloLens (Microsoft Corp, USA), and other devices *via* a wireless network. Upon completion of patient registration, the surgeon could visualize the preoperative STL model in the HoloLens and interact with the hologram using predetermined gesture controls. The surgeon could also adjust the position of the 3D hologram in the surgical field to allow visualization of both the 3D hologram and the surgical plan in real time without having to take hands or eyes away from the surgical field.

### Surgical Process

**Figure 3:**
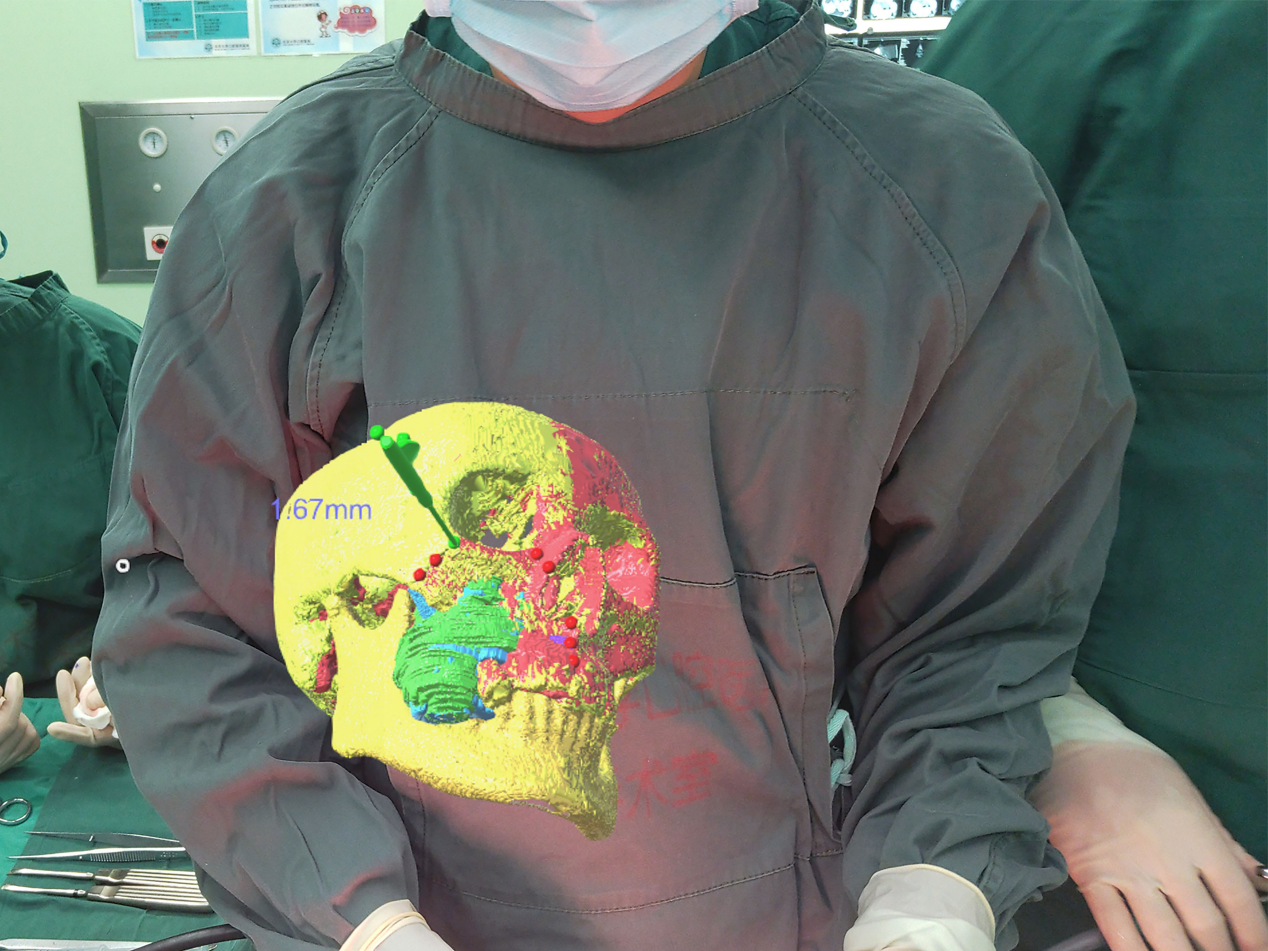
When the tip of the probe was 1.67 mm away from the marked point, the color of the marked point displayed in green.

**Figure 4:**
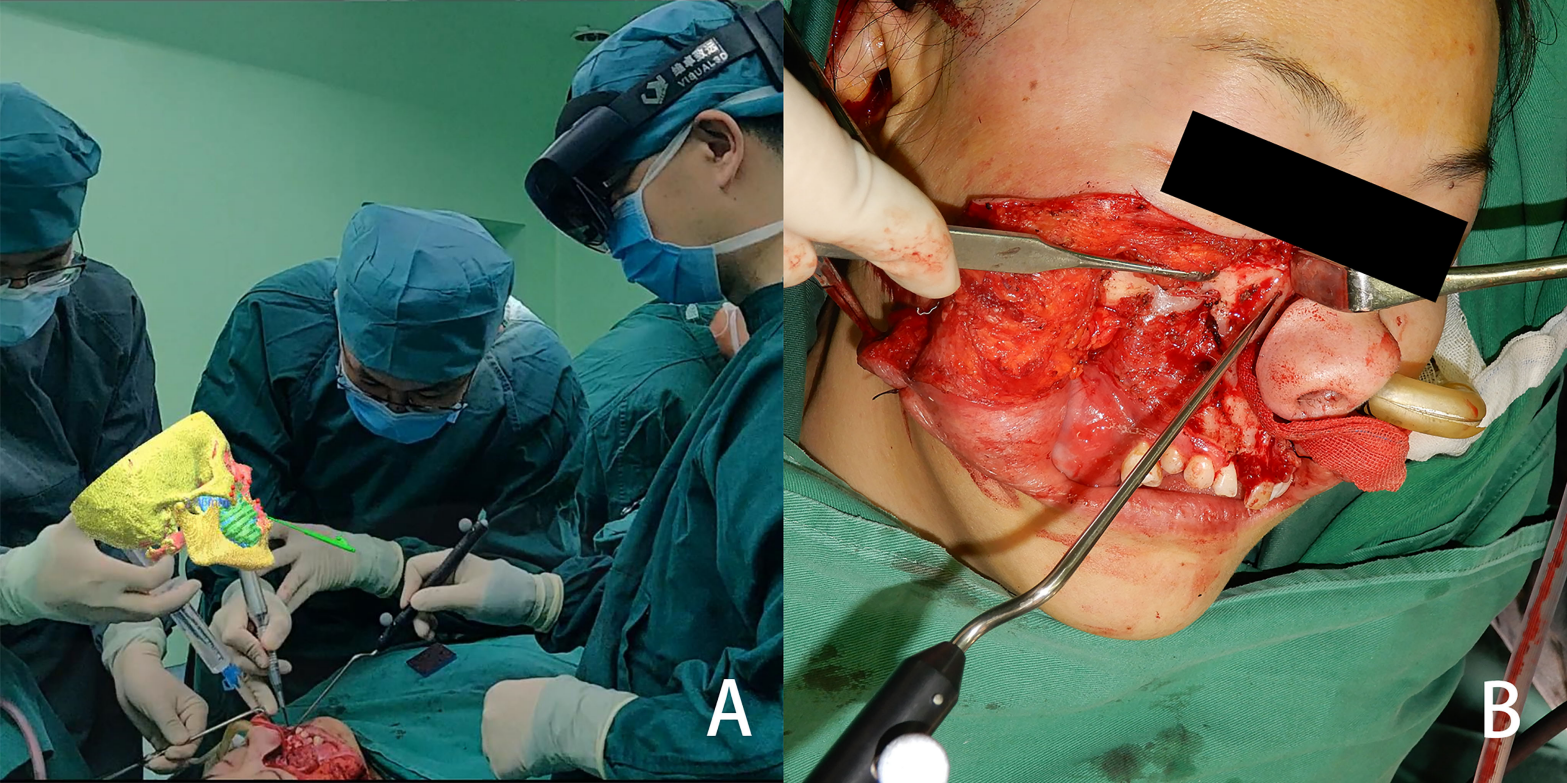
From the view of spectator, the image is located above the surgical area, and the three-dimensional reconstructed image and the actual surgical area are both in the surgeon’s field of view **(A)** the osteotomy line is determined with the help of the mixed reality image **(B)**.

After the tumor was exposed, the surgeon donned the head-mounted HoloLens to facilitate the osteotomy procedure. Using gesture controls, the surgeon manipulated the 3D holographic image—enlarging, shrinking, rotating, hiding or adjusting the transparency as necessary. Before making the osteotomy lines, the surgeon used a hand-held navigation probe to verify the surgical plan through the predetermined reference points on the osteotomy lines. This could be performed without taking the eyes off the surgical field, as the probe pointer and its spatial distance from objects and its relationships were projected in the HoloLens. The allowance for error of the mixed reality system was set at 2 mm, which will lead the surgeon to find the accurate position of the osteotomy lines as soon as possible. The pointer changed color from red to green when the distance between the navigation probe and the reference point was ≤2 mm, thus ensuring the accuracy of the osteotomy lines (**Figure 3**). Through the 3D image and the distances displayed in HoloLens, the surgeon could adjust the direction and position of the hand-held navigation probe and thus verify the osteotomy lines. (**Figure 4**). In patients requiring mandibulectomy, the occlusion was stabilized with maxillo-mandibular fixation, to ensure the relative position of the mandible to the maxilla was the same as in the CT data. The osteotomies and tumor resection were then completed using a reciprocating saw. All surgeries were performed by the same experienced surgeon and his surgical group. Intraoperative frozen section examination was performed in all cases to ensure negative surgical margins.

### Postoperative Evaluation

**Figure 5:**
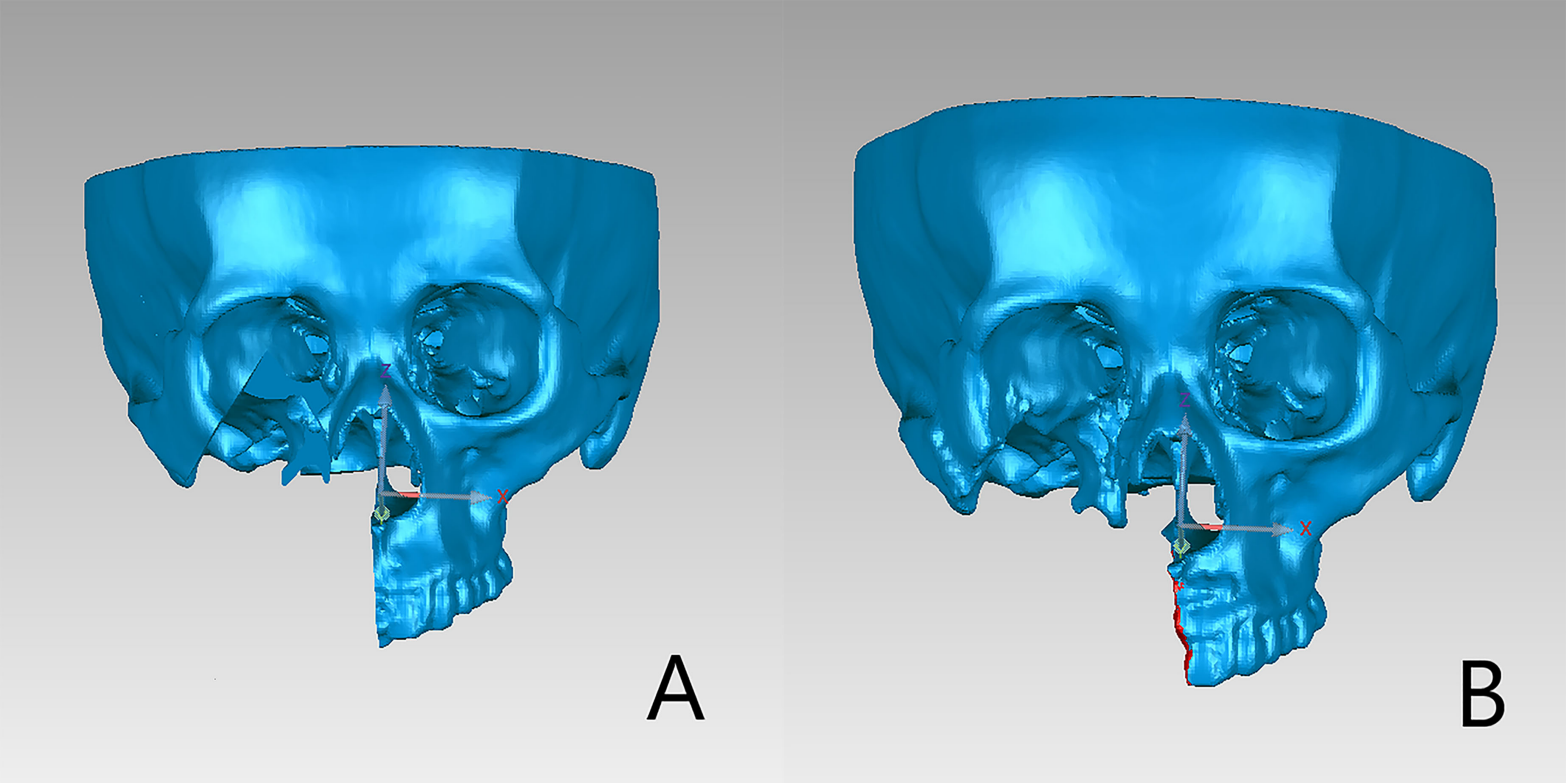
Preoperative and postoperative 3D models included only the remaining maxillary or mandible. **(A)** Preoperative plan. **(B)** Postoperative three-dimensional reconstructed image.

**Figure 6:**
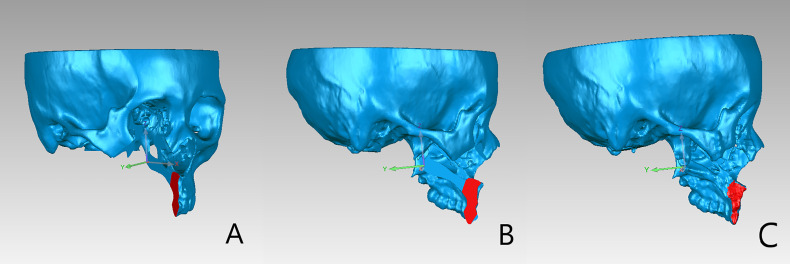
**(A)** The alveolar osteotomy plane was selected on the preoperative maxillary model. **(B)** After clicking “Normal to” button, the view of the models was adjusted as the selected plane was perpendicular to the user’s view of sight. **(C)** The corresponding osteotomy plane on the postoperative model was selected under the adjusted view.

**Figure 7:**
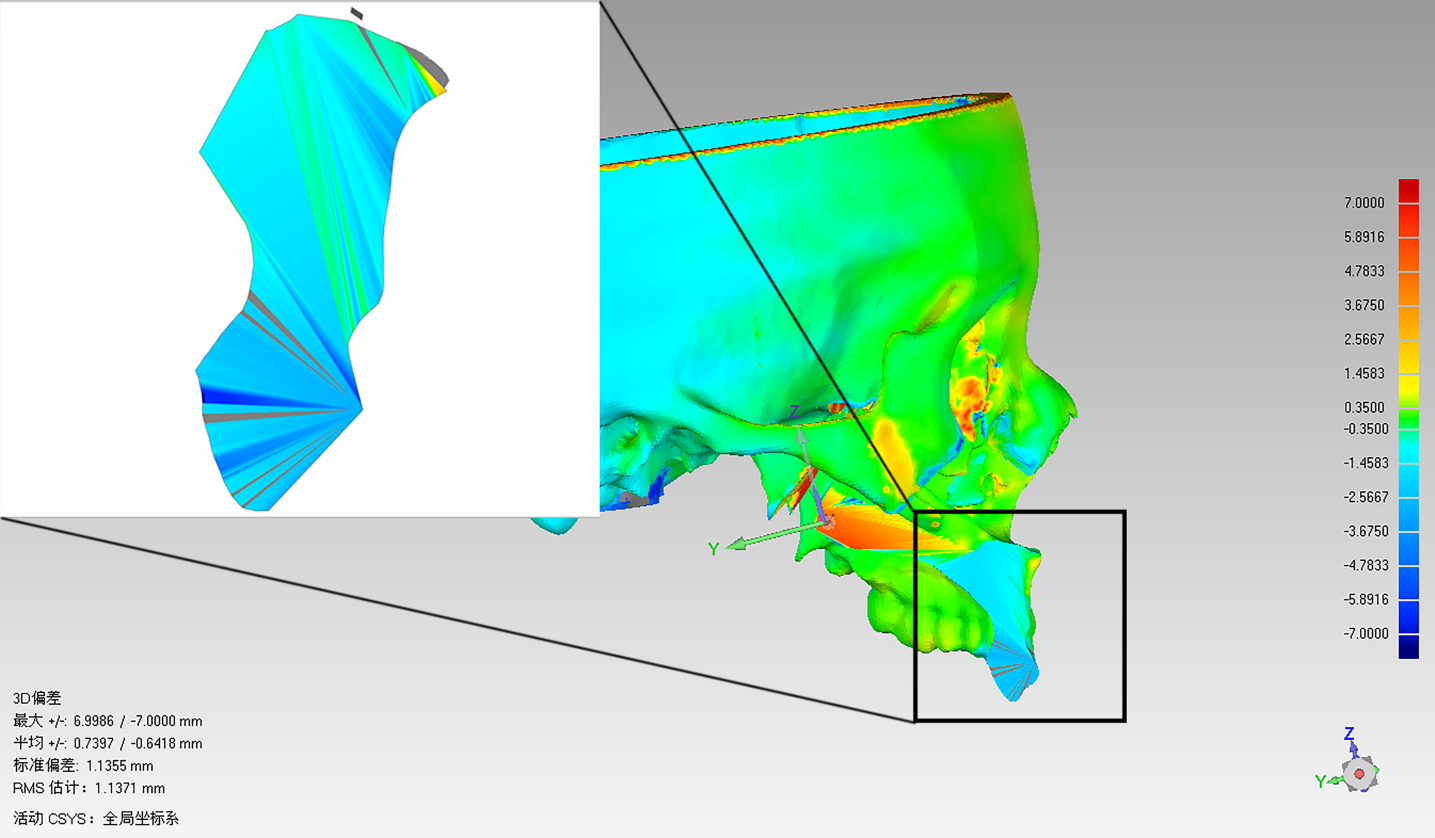
Chromatographic analysis of the maxilla and osteotomy surface using Geomagic software.

Facial CT scan was performed for all patients at 1 week after surgery to evaluate the accuracy of the mandibular, maxillary dentoalveolar, and maxillary-zygomatic osteotomy planes. DICOM data of the postoperative CT scan was imported into ProPlan CMF 3.0 for segmentation and reconstruction of the postoperative 3D models. The preoperative and postoperative 3D models included only the remaining mandible or maxilla and both of them were imported into Geomagic Qualify software (Geomagic, Cary, NC, USA) for accuracy evaluation(**Figure 5**). The preoperative and postoperative 3D models were registered based on the unaffected maxilla and skull base for maxillary tumors, or on the unaffected mandible for mandibular tumors. The operator delineated the osteotomy plane of the maxilla/mandible on preoperative model (**Figure 6A**), then clicked the “Normal To” button and selected one point of the osteotomy plane. “Normal To” function means adjusting a user’s view of an object (but does not modify object’s transformation matrix) so that a selected point appears “closest” to the user. At this time, the osteotomy plane was perpendicular to the user’s view of sight (**Figure 6B**). Within such view, the surface on the postoperative maxilla/mandible model was selected (**Figure 6C**). Accuracy analysis was performed based on the osteotomy planes of the selected areas (7). The program automatically recognized the corresponding points from the two areas and calculated the distance between the corresponding points, then mean deviation and color map were computed automatically (**Figure 7**). All patients received standard oncological follow-up for at least 6 months postoperatively. Tumor recurrence and/or postoperative complication(s) (if present) were recorded during review. All data analyses were conducted using SPSS 21.0 (IBM Corp, Armonk, NY, USA).

## Results

Among the seven patients enrolled in this study (**Table 1**), four had maxillary tumors and three had mandibular tumors. The median age of the patients was 45 years. There were a total of 13 groups of osteotomy planes. The mean deviation between preoperative virtual osteotomy plane and actual postoperative osteotomy plane was 1.68 ± 0.92 mm, with the largest deviation being 3.46 mm. Color map analysis showed that 80.16% of mean deviations from the actual osteotomy surface was within 3 mm. The mean deviations of maxillary and mandibular osteotomy planes were approximate (1.60 ± 0.93 mm vs. 1.86 ± 0.93 mm). Of the seven patients, five had benign tumors and two had malignant tumors. The mean deviations of osteotomy planes of patients with benign and malignant tumors were comparable (1.48 ± 0.74 mm vs. 2.18 ± 0.77 mm). Intraoperative frozen section biopsy confirmed negative margins in all cases. Mean follow-up was for 15.7 months (range, 6-26 months). The postoperative course was uneventful in all patients, and no patient had tumor recurrence or major complications during follow-up.

**Table 1:** Basic data and follow-up outcomes of the seven patients.

Patient	Sex	Age, years	Lesion Range	Pathology	Complications	Follow-Up Time, Months
1	Male	34	Mandibular	Ameloblastoma	No	26
2	Male	45	Maxilla	Ameloblastic fibro-odontoma	No	25
3	Male	19	Maxilla	Odontogenic myxoma	No	22
4	Male	69	Maxilla	Osteosarcoma	No	16
5	Male	55	Mandibular	Squamous cell carcinoma	No	8
6	Female	37	Maxilla	Odontogenic myxoma	No	7
7	Female	50	Mandibular	Ossifying fibroma	No	6

## Discussion

Surgery remains the mainstay of management for neoplasms in the oral and maxillofacial region (8). Surgical resection with negative surgical margin can be challenging, particularly for deep-seated tumors. Accurate and safe tumor resection requires thorough understanding of the tumor site and tumor size, characteristics, and margins. CT and MRI are important tools for diagnosing and staging neoplasms (9). While CT scan is especially useful for identifying tumors of bony origin, MRI is better for evaluation of soft tissue masses. The combination of the two modalities provides comprehensive assessment of tumor extension and its relationship with adjacent vital structures.

The CAS technique, which includes 3D image reconstruction, computer-aided design and computer-aided manufacturing (CAD/CAM), surgical navigation, and robotic surgery, has been widely applied in the field of oral and maxillofacial surgery in the past decade to improve surgical accuracy and final outcomes (2). With the image fusion technique, CT and MRI data can be combined to provide highly informative data for accurate diagnosis and treatment planning (10). CAD/CAM cutting guides permit accurate translation of virtual surgical planning to the surgical field (11). However, the surgical guides or plate may not be located easily and the incisions needed to be extended leading to enlarged damage to the normal tissue (2).

Several studies have shown surgical navigation to be a cost-effective and efficient method for translating the virtual surgical plan into operative reality and enhancing surgical accuracy and safety. Andrews et al. (12) used surgical navigation for orbital reconstruction and found that implant position can be verified using intraoperative navigation, thus reducing the risk of optic nerve injuries. Zhang et al. (13) applied surgical navigation in maxillary tumor resection and orbital reconstruction, and were able to accurately restore orbital volume, without encountering postoperative complications such as diplopia, restriction of ocular movements, or impaired visual acuity. Inthecurrentstudy (5), the registration accuracy of surgical navigation using a dynamic reference frame and laser surface scanning was ≤1 mm, and the overall surgical accuracy was ≤2 mm.

Current intraoperative navigation systems enable surgeons to inspect and interact with 3D images and objects on a flat-panel display (14). For realistic images, optimal contrast and depth perception is essential, but conventional image presentation on a 2D screen cannot provide spatial relationship and depth information effectively. Distractions due to any cause (e.g., reorientating of the radiographic images, the surgical plan, or equipment issues) during surgical procedures can be deleterious (15). With the holographic imaging technology, it is now possible for users wearing a head-mounted display to manipulate and interact with virtual objects in real time (16). In this study, HoloLens was used as the display unit to project holograms onto the surgical field; this reduced the distraction of surgeons when viewing the surgical navigation plane display.

In previous studies on mixed reality, the superimposition method was used to overlay holograms over actual anatomical structures, and manual matching was then performed. Zhu et al. (17) matched the reference points in the surgical field to the reference points in the 3D image. Mitsuno et al. (18) introduced a new and fast mechanism of alignment; they matched corresponding reference points in the hologram and the actual surgical field, and the mean time for alignment within 50 seconds, with mean error controlled to within 3 mm. The main drawbacks of manual matching are the time-consuming matching process and the low matching accuracy. Li et al. (19) used mixed reality technology to guide external ventricular drain insertion, and reported a mean deviation of 4.34 ± 1.63 mm and additional preoperative preparation time of 40.20 ± 10.74 minutes. It must be noted that in previous research (18–20), the manual matching was applied in the process of adjustment of hologram. Under such circumstance, the spatial position of the hologram was fixed. The physical movements of the surgical field will require re-adjustment of the hologram and the surgical field to keep the hologram consistent with the object in the surgical area. These issues prevent wider application of mixed reality in surgical procedures.

To the best of our knowledge, the application of mixed reality technology plus surgical navigation has not been previously reported in oral and maxillofacial surgery. This study used IGT-link port to connect the intraoperative navigation and mixed reality workstations, thus enabling projection of holograms on to the surgical field through HoloLens in real-time. It has the following advantages (1): The tumor, with the important surrounding structures and the virtual surgical plan can be visualized in real time, helping the surgeon to notice and protect them during the operation. (2) The real-time display of the distance between the marker point and the probe tip can help the surgeon determine the position of the osteotomy line efficiently. (3) Hololens supports gesture operation, so the surgeon can manipulate the 3D hologram (translation or rotation) without touching the object.

The evaluation of accuracy was carried out by two oral and maxillofacial surgeons, and each surgeon repeated the measurement twice. The final result was the average of four values. The intraclass correlation was also calculated to check the repeatability of evaluation method. In our patients, the mean deviation between virtual and actual osteotomy planes was 1.68 ± 0.92 mm. The mean deviation was larger for mandibular osteotomy planes than for maxillary osteotomy planes (1.86 ± 0.93 mm vs. 1.60 ± 0.93 mm), likely due to the mobile nature of mandible. Huang et al. (20) incorporated surgical navigation in mandibular reconstruction with free fibula flap and reported a mean deviation of 4 mm between preoperative virtual surgical plan and the actual length, width, and height of the reconstructed mandible. Casap et al. (21) compared two surgical navigation systems and noted lower navigational error with dental implants navigation system (<0.5 mm) than with otorhinolaryngology navigation system that used a head-mounted reference frame (~3-4 mm). In our own previous study (22), we integrated personalized cutting guides and intraoperative navigation system in mandibular reconstruction and found a mean deviation of 2.017 ± 0.910 mm. In the present study, the direct visualization of mandibular hologram in the surgical field with the use of mixed reality, and the maxillo-mandibular fixation, both helped reduce deviation during mandibulectomy.

In navigation-assisted surgery, surgical efficiency and accuracy is adversely affected by the need for the surgeon to verify the surgical plan repeatedly using a hand-held navigation probe on axial, sagittal, and coronal images displayed on a flat-panel screen. HoloLens, by projecting the image directly on to the surgical field, largely avoids the need for shifting gaze. The distance between the hand-held navigation probe and the actual reference point is continuously displayed with color and numerical indicators and thus increase the efficiency and safety of the surgery.

Previous authors have pointed out several issues with the use of HoloLens. One important problem is visual discrepancy. The hologram may appear in a different spatial position in the assistant’s view, even after registration by the surgeon. Galati et al. (14) reported a discrepancy of 4.5 cm when the same reference point was viewed from different perspectives. Visual discrepancy is potentially dangerous. Another problem that has been reported is that overlap of the hologram with the surgical field may obstruct the view of anatomical structures. The operating light might also affect the quality of the hologram (23). In our patients, we overcame the problem of visual discrepancy by using mixed reality in combination with surgical navigation (to establish the coordinates). Since the structure in hologram could be displayed real-time by surgical navigation, the surgeons could place the hologram at anywhere in his/her field of view, which meant it was not necessary that the hologram overlapped with the corresponding surgical field. The surgeons could translate or rotate the hologram to a proper place where the surgeons could simultaneously get the sight of hologram and the surgical field. This could help to reduce the disturbance caused by the overlapping between hologram and surgical field.

Mixed reality technology has its limitations. The HoloLens device is relatively bulky and heavy, and wearing it for long periods can be uncomfortable. Although mixed reality technology provides hologram display in real-time, the registration and “passive verification” still relies on navigation system. It required additional steps to combined mixed reality with surgical navigation, including equipment connection, fixation of dynamic reference frame and surgical navigation registration, which may require more preparation time.

This study was to explore the feasibility of combining navigation system with mixed reality in surgery. Accuracy is an aspect of evaluating the application effect of this technique. Our research team has proved the accuracy of surgical navigation in previous studies (13, 24). Whether the combination of mixed reality and surgical navigation was more accurate than single surgical navigation remains to be solved in the future. After verifying the feasibility of mixed reality combined with surgical navigation, we will set up a control group in which tumors are resected under the guidance of only surgical navigation.

## Conclusion

The combination of mixed reality technology and surgical navigation appears to be safe and effective for tumor resection in the oral and maxillofacial region. The two technologies have complementary advantages. However, further research is needed to validate the application of surgical navigation in the mixed reality environment.

## Data Availability Statement

The original contributions presented in the study are included in the article/supplementary material. Further inquiries can be directed to the corresponding authors.

## Ethics Statement

The studies involving human participants were reviewed and approved by the Institutional Review Board of Peking University School of Stomatology. The patients/participants provided their written informed consent to participate in this study. Written informed consent was obtained from the individual(s) for the publication of any potentially identifiable images or data included in this article.

## Author Contributions

Z-NT, W-BZ, and YY designed the study. Z-NT, L-HH, and HS collected the data and performed the statistical analysis. Z-NT, HS, and W-BZ wrote and revised the manuscript. W-BZ and XP contributed to the conception and design of the study. All authors have approved the final version to be published.

## Funding

This work was supported by Program of the new clinical techniques of Peking university school and hospital of stomatology(PKUSSNCT-20A05).

## Conflict of Interest

The authors declare that the research was conducted in the absence of any commercial or financial relationships that could be construed as a potential conflict of interest.

## Publisher’s Note

All claims expressed in this article are solely those of the authors and do not necessarily represent those of their affiliated organizations, or those of the publisher, the editors and the reviewers. Any product that may be evaluated in this article, or claim that may be made by its manufacturer, is not guaranteed or endorsed by the publisher.
